# Double-antigen sandwich ELISA based on chimeric antigens for detection of antibodies to *Trypanosoma cruzi* in human sera

**DOI:** 10.1371/journal.pntd.0010290

**Published:** 2022-03-11

**Authors:** Natália Erdens Maron Freitas, Emily Ferreira Santos, Leonardo Maia Leony, Ângelo Antônio Oliveira Silva, Ramona Tavares Daltro, Larissa de Carvalho Medrado Vasconcelos, Gabriela Agra Duarte, Cristiane Oliveira da Mota, Edimilson Domingos Silva, Paola Alejandra Fiorani Celedon, Nilson Ivo Tonin Zanchin, Fred Luciano Neves Santos

**Affiliations:** 1 Gonçalo Moniz Institute, Oswaldo Cruz Foundation (FIOCRUZ-BA), Salvador, Bahia, Brazil; 2 Gonçalo Moniz Public Health Central Laboratory (LACEN-BA), Salvador, Bahia, Brazil; 3 Bio-Manguinhos, Oswaldo Cruz Foundation (FIOCRUZ-RJ), Rio de Janeiro, Rio de Janeiro, Brazil; 4 Molecular Biology Institute of Paraná (IBMP), Curitiba, Paraná, Brazil; 5 Carlos Chagas Institute, Oswaldo Cruz Foundation (FIOCRUZ-PR), Curitiba, Paraná, Brazil; 6 Integrated Translational Program in Chagas disease from FIOCRUZ (Fio-Chagas), Oswaldo Cruz Foundation (FIOCRUZ-RJ), Rio de Janeiro, Rio de Janeiro, Brazil; University of Texas at El Paso, UNITED STATES

## Abstract

**Background:**

Enzyme-linked immunosorbent assays (ELISA) are generally the chosen test for Chagas disease (CD) diagnosis; however, its performance depends on the antigen preparation adsorbed to the solid phase, which may lead to false-positive results and cross-reactions. The use of chimeric recombinant antigens can overcome this limitation. Four chimeric antigens from *Trypanosoma cruzi* (IBMP-8.1, IBMP-8.2, IBMP-8.3 and IBMP-8.4) were developed and evaluated in phase I, II and III studies using indirect ELISA as diagnostic platform. However, peroxidase-labeled secondary anti-human IgG antibody, which is employed in indirect ELISAs, limits its use for the detection of species-specific and class-specific antibodies. To overcome this limitation, peroxidase-labeled antigens can be utilized, diagnosing both acute or chronic infection, in a species and immunoglobulin class-independent manner, through the use of a double-antigen sandwich ELISA (DAgS-ELISA). We aimed to evaluate and validate the diagnostic performance of the chimeric antigens IBMP-8.1, IBMP-8.2, IBMP-8.3 and IBMP-8.4 in the DAgS-ELISA platform.

**Methodology/Principal findings:**

DAgS-ELISA was optimized by checkerboard titration. In phase I study, 207 positive and 205 negative samples were evaluated. Cross-reactivity to other infections was also assessed using 68 samples. The selected conditions for the tests utilized 25 ng of antigen per well and the conjugate diluted at 1:2,000 for all molecules. In the phase I study, the areas under the curve of IBMP-8.1, IBMP-8.2, IBMP-8.3 and IBMP-8.4 were 98.7%, 99.5%, 98.6% and 98.8%, respectively. Among the positive samples, IBMP-8.1 antigen classified 53 (25.6%) as false negative, IBMP-8.2, 27 (13%), IBMP-8.3, 24 (11.6%) and IBMP-8.4, 43 (20.8%), giving sensitivities of 74.4%, 87%, 88.4% and 79.2%, respectively. The only antigen that did not reach 100% specificity was IBMP-8.3, with 96.6%. IBMP-8.3 was also the only molecule to show cross-reactivity with HTLV.

**Conclusions/Significance:**

DAgS-ELISA is a promising tool for immunodiagnosis, and despite the high AUC values, the performance of this assay was different from the values obtained by our group when using these antigens in the indirect ELISA, for this reason, improvements are being considered to increase the sensitivity of the DAgS-ELISA.

## Author summary

Indirect immunoassays are the recommended method for chronic Chagas disease diagnosis and its performance relies on the employed antigen preparation. Chimeric antigens have been successfully utilized for chronic CD in vitro diagnosis and efficiently addresses commonly encountered hurdles arising from the use of recombinant and native antigens. Thus, our group developed four *Trypanosoma cruzi* chimeric antigens and evaluated their diagnostic potential in an indirect Enzyme-linked immunosorbent assays (ELISA) design; however, this design restricts detection to a single antibody class and for a single species. To overcome this, we utilized conjugated antigens in a double-antigen sandwich ELISA (DAgS-ELISA) design, which is able to detect both acute and chronic infections, in a species and immunoglobulin class-independent manner. Despite their high discrimination levels, as illustrated through elevated AUC values, our results were different from those obtained previously, especially regarding sensitivity. Specificity values remained elevated, reaching 100% for 3 out of 4 antigens. As such, improvements are being considered to increase the sensitivity of the DAgS-ELISA.

## Introduction

Chagas disease (CD), also known as American trypanosomiasis, is a potentially life-threatening vector-borne tropical zoonosis caused by the protozoan parasite *Trypanosoma cruzi*. The estimated prevalence of CD in 21 Latin American countries where it is considered endemic exceeds five million individuals; CD accounts for approximately 7,500 deaths annually [1]. As a result, CD is considered a relevant neglected disease in the Americas: only approximately 7% of CD-infected people have been diagnosed, with far fewer (~1%) receiving etiological treatment [2,3]. Infection may occur through contact with the feces or urine of infected vectors, blood transfusion, organ transplantation, consumption of contaminated food and beverages and vertical transmission, as well as laboratory accidents. Since the late 1990s, CD has become increasingly recognized as a public health problem in Europe, Australia, Japan and the US due to human migratory flows [4,5].

CD has an acute phase, which can last up to a few weeks or months, and usually presents as an unspecific or asymptomatic self-limiting febrile illness [6]. The acute phase is characterized by high-grade parasitemia; therefore, direct microscopic observation is the preferred method of diagnosis. If left untreated, *T*. *cruzi*-infected individuals enter a lifelong chronic phase. Decades after becoming infected, an estimated 30–40% of chronically infected individuals will develop life-threatening, debilitating medical problems characterized by severe cardiac, digestive, neurological or mixed alterations [6,7]. Due to a scarcity or lack of circulating parasites and high anti-*T*. *cruzi* IgG antibody titers, diagnosis during the chronic stage relies on the utilization of antibody-antigen methods, including enzyme-linked immunosorbent assay (ELISA), indirect hemagglutination assay (IHA), indirect immunofluorescence (IIF), point-of-care tests (POC) and chemiluminescent assays (CLIA). Due to its simplicity and ease of automation, ELISA has become the most common indirect serological assay for chronic CD diagnosis worldwide.

The diagnostic performance of ELISA in detecting anti-*T*. *cruzi* antibodies is particularly dependent on the antigen preparations used to sensitize the solid phase of the microplate immunoassay. Parasite lysate and whole-cell *T*. *cruzi* homogenate have been used as complex antigen mixtures, rendering high sensitivity values at the expense of low specificity and high rates of cross-reactivity [8,9]. Conversely, recombinant *T*. *cruzi* proteins have been shown to increase specificity but decrease sensitivity due to their limited epitope repertoire [10,11]. The use of recombinant chimeric antigens is one strategy to overcome the irregular diagnostic performance of serological assays. These molecules are composed of repetitive and conserved tandem motifs of epitopes from several antigenic parasite proteins [10,12].

The diagnostic performance of four *T*. *cruzi* chimeric recombinant proteins (IBMP-8.1, IBMP-8.2, IBMP-8.3 and IBMP-8.4) has been extensively evaluated using anti-human IgG or anti-dog IgG-peroxidase antibodies in the serologic diagnosis of CD (using ELISA, liquid microarray, lateral flow assay, or surface plasmon resonance) in humans residing in both endemic [9,13–17] and non-endemic settings [15], as well as in dogs [18]. Despite achieving high performance, this approach is limited to specific species, thus requiring differently labeled antibodies in accordance with sample origin.

Double-antigen sandwich ELISA (DAgS-ELISA) is a platform offering high sensitivity that has been used to detect antibodies against different pathogens [19–23], regardless of class and species [19,24,25]. We developed a strategy involving the use of peroxidase-labeled soluble IBMP antigens to detect antigen-antibody complexes formed during immunoassays. The present report consists of a proof-of-concept study (phase I) to assess the diagnostic performance of these four IBMP chimeric proteins using a DAgS-ELISA platform to detect anti-*T*. *cruzi* antibodies in human samples.

## Material and methods

### Ethics statement

The Institutional Review Board (IRB) for Human Research at the Gonçalo Moniz Institute of the Oswaldo Cruz Foundation (IGM-FIOCRUZ), Salvador, Bahia-Brazil approved this study (CAAE: 67809417.0.0000.0040).

### Chimeric antigens synthesis

The chimeric antigens used herein were obtained according to Santos et al. [12]. Briefly, the antigens were cloned into pET28a vectors, expressed in *Escherichia coli* BL21-Star (ThermoFisher Scientific), with expression induced using 0.5 μM of IPTG (isopropyl β-D-1-thiogalactopyranoside). Bacteria were lysed and the homogenate containing the proteins was centrifuged for purification by ion exchange and affinity chromatography.

### Peroxidase labeling process

The peroxidase labeling process was carried out in collaboration with the Laboratory of Diagnostic Technology, Bio-Manguinhos (FIOCRUZ-RJ). Chemical conjugation was performed using the hydrazone method [26,27]. In this process, sodium metaperiodate is used as an oxidizing agent for peroxidase. Sodium borohydride is responsible for the formation of the hydrazone bond between peroxidase and the chimeric antigen. To obtain an activated form, 3 mg HRP in 0.5 mL deionized water was mixed with 0.5 mL 0.1 M sodium periodate in sodium phosphate buffer (pH 7.0), and the mixture was incubated for 20 minutes at room temperature (RT; 25 ± 3°C) in the dark. The activated HRP was then dialyzed in 3 liters of 1 mM sodium acetate (pH 4.4) overnight at 4°C. Meanwhile, the IBMP proteins, which had been prepared in 50 mM Tris-buffered NaCl solution (pH 8.0) for IBMP-8.4 and 150 mM phosphate-buffered solution (pH 6.5) for IBMP-8.1, IBMP-8.2, and IBMP-8.3 solutions, were buffer-exchanged in 0.34 mL 50 M carbonate- bicarbonate (pH 9.5) using a 10 K-Cutoff Amicon Ultra-0.5 centrifugal filter device (EMD Millipore, Billerica-MA, USA) according to the manufacturer’s protocol. The dialyzed IBMP antigens were then mixed with the dialyzed HRP solution, and the mixture was incubated for 2 hours at RT in the dark. The conjugate was stabilized by adding 0.1 M sodium borohydride (0.25 mL for 1 mg IBMP protein) to the reaction solution and incubated for an additional 2 hours at 4°C. Subsequently, the mixtures were buffered in 3 mL of a 0.01 mM phosphate-buffered solution (pH 7.2) using a 10 K-Cutoff Amicon Ultra-0.5 centrifugal filter device. Finally, a stabilizing solution (HRP-StabilPlus; Kem-En-Tec, Taastrup, Denmark) was added to the resulting solution (1:2) and the IBMP-labeled HRP proteins were stored at -20°C.

### Sample collection

Sample size was computed for an infinite population with a 95% confidence interval, an absolute error of 1.4%, and an expected sensitivity and specificity of 99%. Based on these parameters, obtained using OpenEpi, a free web-based open-source program [28], the minimum sample to perform this study was 196 sera each from *T*. *cruzi*-positive and -negative individuals. A total of 412 sera were obtained from *T*. *cruzi*-positive (n = 207) and *T*. *cruzi*-negative individuals (n = 205), originating from the Central Public Health Laboratory of the State of Bahia (LACEN/BA) and the Hematology and Hemotherapy Foundation of Bahia (HEMOBA), respectively. Additionally, to evaluate cross-reactivity, 68 sera from individuals with unrelated diseases, as previously defined by parasitological or serological diagnosis, were obtained from the sera banks of HEMOBA and the Federal University of Rio Grande do Norte: leishmaniasis (n = 10), hepatitis B (n = 20), hepatitis C (n = 10), HTLV-1/2 (n = 9), HIV-1/2 (n = 9) and syphilis (n = 10). All sera provided by HEMOBA were screened via Liaison XL Murex CLIA. Samples were retested only if the resulting reactivity index (RI) was equal to or greater than 0.75 and a new blood sample was required for retesting. Conversely, the blood sample was considered negative if the reactivity fell below 0.75. In LACEN/BA, sera were tested using two techniques: indirect recombinant ELISA and chemiluminescence, as recommended by WHO. All sera submitted to DAgS-ELISA had been previously retested to detect *T*. *cruzi*-antibodies by indirect ELISA using the four chimeric IBMP antigens combined with a Latent Class Analysis (LCA) statistical approach as a reference test for Chagas disease [29]. Briefly, LCA defined a given sample as *T*. *cruzi*-positive when at least two of the four chimeric antigens yielded positive results (posteriori probability ≥ 88%). Conversely, a sample was classified as negative for *T*. *cruzi* if at least three of the four chimeric antigen-based assays yielded negative results (posteriori probability ≤ 0.8%). Results from each IBMP ELISA were expressed in index format and then plotted to represent the ratio between the OD of a given sample and the cutoff value (CO) of optical density (OD) for each microplate. Index values are referred to as reactivity index (RI); all results < 1.00 were considered negative. In addition, a commercial indirect ELISA (Gold ELISA Chagas (Rem Diagnóstica, São Paulo, Brazil), which uses both recombinant antigens and purified lysates from Brazilian strains of *T*. *cruzi* epimastigotes) was used to re-characterize the serum panel. Therefore, samples were considered seropositive for *T*. *cruzi* if they were reactive with both the LCA and Gold ELISA Chagas. All commercial assays were performed in strict accordance with the manufacturer’s specifications. A unique identifier code was applied to each sample to ensure a blinded analysis. Individual data points for all assays used by HEMOBA, LACEN-BA, and FIOCRUZ are provided in S1 Table. The institutions that provided the samples did not provide information on the severity of the disease or the clinical form.

### Double-antigen sandwich ELISA optimization and procedure (IBMP-DAgS-ELISA)

The optimal dilutions of serum and antigen-enzyme conjugate (HRP) were determined by checkerboard titration at different chimeric antigen coating concentrations. Antigens were diluted at the final concentrations of 400 ng, 200 ng, 100 ng, 50 ng, 25 ng, 12.5 ng, 6.25 ng, 3.125 ng and 1.56 ng in carbonate-bicarbonate buffer (50 mM, pH 9.6). These dilutions (100 μL) were placed on 96-well high-binding microplates (UV-Star Microplate, Greiner Bio-One, Kremsmünster, Austria). After a blocking step with WellChampion (Kem-En-Tec, Taastrup, Denmark), 50 μL of each sera sample, diluted 1:2 or not in phosphate-buffered saline (PBS; pH 7.4), was added, followed by incubation at 37°C for 60 minutes. The plates were then washed with phosphate-buffered saline-0.05% Tween 20, and 50 μL of the HRP-labeled antigens was added at dilutions of 1:500, 1:1,000, 1:2,000, 1:5,000 and 1:10,000, followed by incubation at 37°C for 30 minutes. After a second washing step, reactions were revealed by adding 100 μL of TMB plus substrate (tetramethyl-benzidine; Kem-En-Tec, Taastrup, Denmark). After 10 minutes of incubation at room temperature in the dark, the reaction was stopped using 50 μL of 0.3M H_2_SO_4_. Absorbance was measured on a microplate spectrophotometer at a wavelength of 450 nm (SPECTRAmax 340PC, San Jose, CA, EUA).

### Statistical analysis

**Fig 1:**
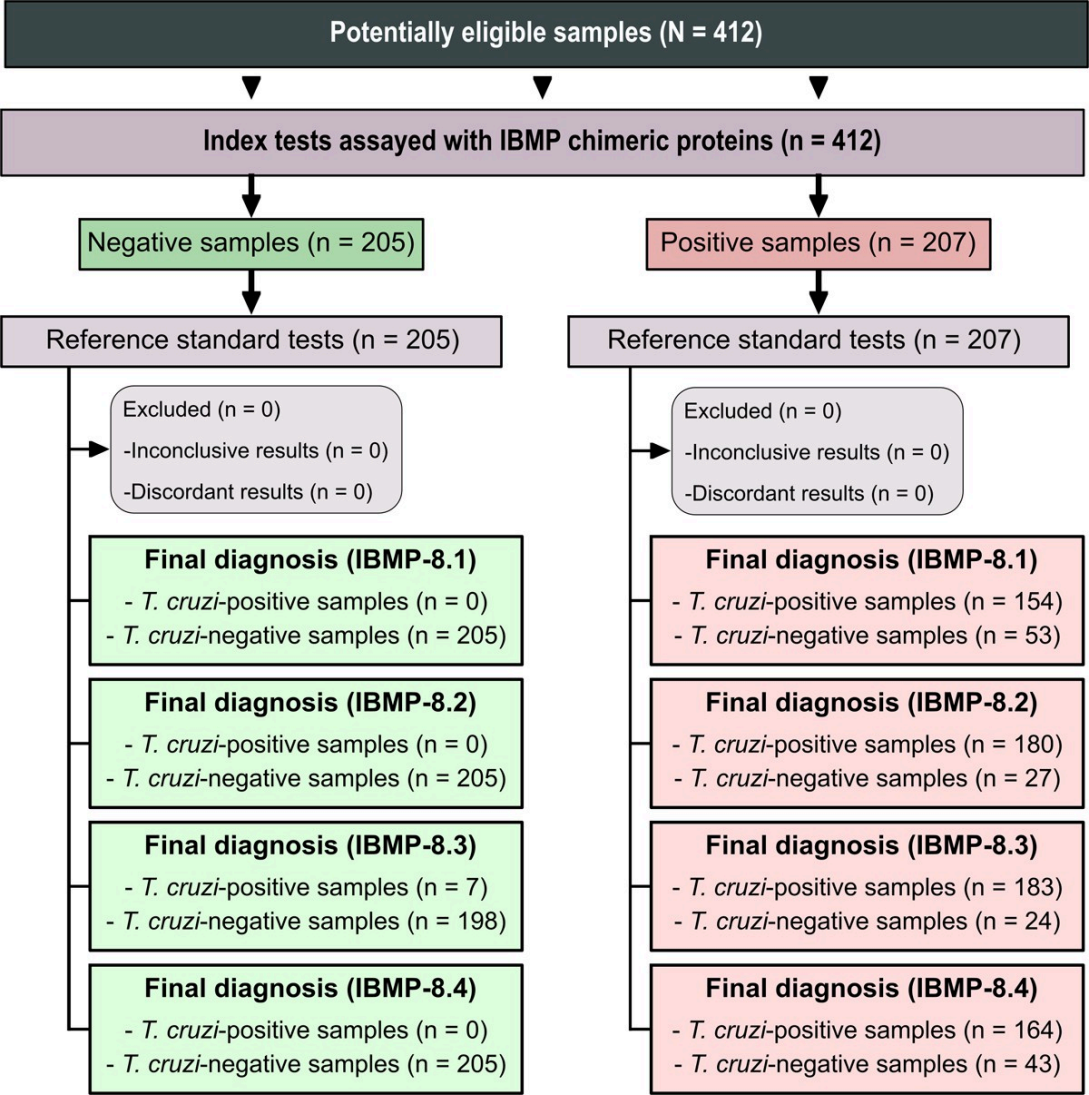
Flowchart illustrating study design in accordance with the Standards for Reporting of Diagnostic Accuracy Studies (STARD) guidelines.

Data were analyzed using graphics plotting software (GraphPad Prism version 8, San Diego, CA, USA). The normality of data was tested using the Shapiro-Wilk test. When the assumed homogeneity was confirmed, Student’s t-test was employed. Wilcoxon’s signed-rank test was used for non-normal distributions. Variables were expressed as arithmetic and geometric means, standard deviations and variation coefficients. All results were evaluated considering a statistical significance level of p <0.05. Cut-off values were established by determining the greatest area under the receiver operating characteristic (ROC) curve (AUC) in order to differentiate between *T*. *cruzi*-positive and negative samples [30]. For this purpose, the same set of positive (n = 10) and negative (n = 10) samples was used in all microtiter plates. Areas under the ROC curve (AUC) were considered to assess the global accuracy for each IBMP chimeric protein, which was classified as low (0.51–0.61), moderate (0.62–0.81), elevated (0.82–0.99), or outstanding (1.0) [31]. Results were expressed as reactivity index (RI) values representative of the ratio between a sample’s optical density (OD) and the cut-off (CO) value established for each corresponding microplate. RI values were interpreted as follows: negative (RI < 1.0), positive (RI ≥ 1.0), and grey zone (0.90 ≤ RI ≤ 1.10). IBMP-DAgS-ELISA performance parameters were compared with respect to sensitivity (Sen), specificity (Spe) and accuracy (Acc). A 95% confidence interval (95% CI) was calculated to determine the precision of the obtained estimates. To better evaluate the diagnostic performance of the four IBMP chimeras, multiple tests (series and parallel approaches) were applied to individual test characteristics. Multiple tests may be ordered at the same time (parallel tests), in which case a positive result in any of the tests is evidence of disease, or they may be ordered sequentially (tests in series), as new tests are requested depending on the result of the previous test. In this case, all results must be positive to establish a diagnosis of disease [32]. The strength of agreement between previously conducted diagnostic assessments and IBMP-DAgS-ELISA was analyzed by Cohen’s *kappa* (κ) index, with values interpreted as poor (κ ≤ 0), slight (0.20 ≥ κ > 0), fair (0.40 ≥ κ ≥ 0.21), moderate (0.60 ≥ κ ≥ 0.41), substantial (0.80 ≥ κ ≥ 0.61) and almost perfect agreement (1.0 ≥ κ ≥ 0.81) [33]. A checklist (S1 Checklist) and a flowchart (Fig 1) are provided according to the Standards for the Reporting of Diagnostic accuracy studies (STARD) guidelines [34].

## Results

### IBMP-DAgS-ELISA optimization

Optimal assay conditions were established considering (1) the largest difference in average OD values between *T*. *cruzi*-positive and negative samples; (2) average OD < 0.150 for *T*. *cruzi*-negative samples; (3) average OD > 0.800 for *T*. *cruzi*-positive samples. Accordingly, a dilution rate of 1:2,000 for HRP-labeled antigens and 25 ng of each IBMP chimeric protein were used to sensitize microplates, with no dilution of serum samples.

### IBMP-DAgS-ELISA performance

**Fig 2:**
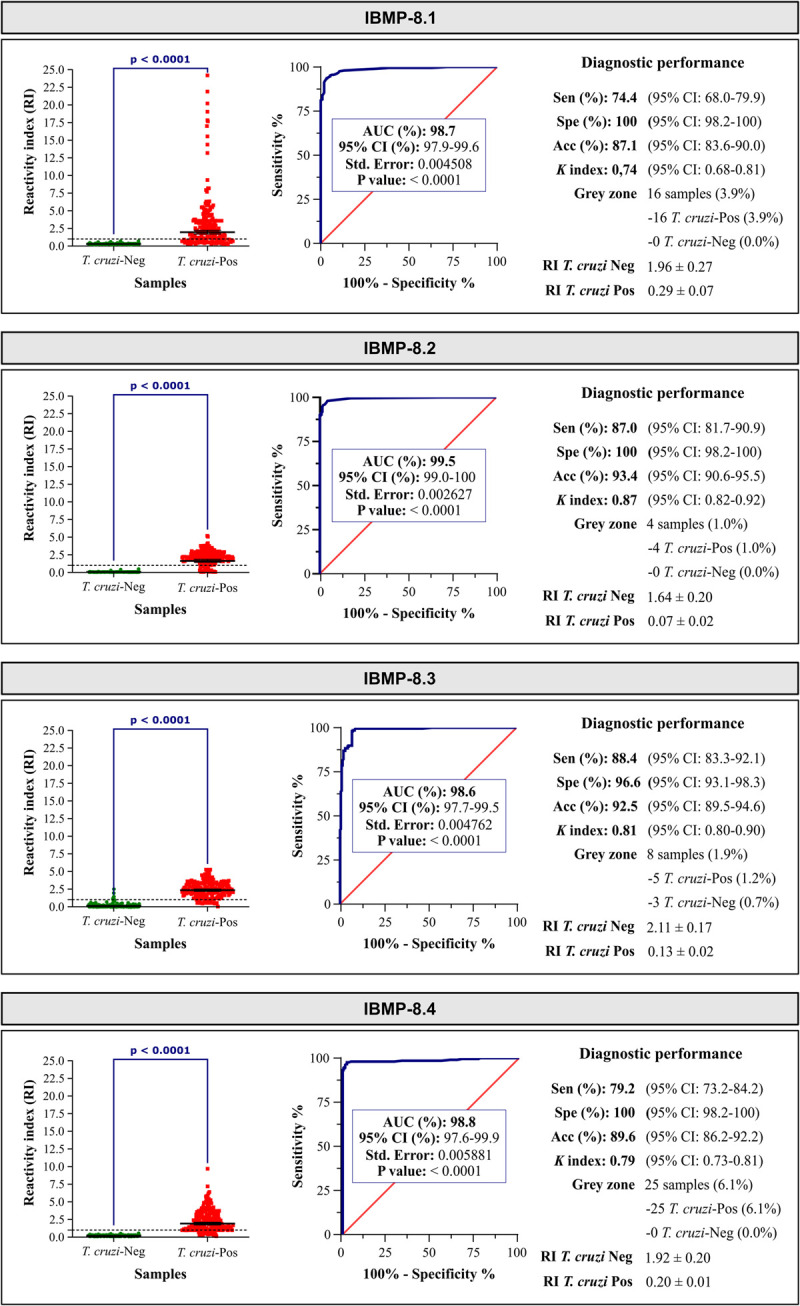
Reactivity Index (RI) and diagnostic performance parameters obtained from *Trypanosoma cruzi*-positive (*T*. *cruzi*-Pos) and *Trypanosoma cruzi*-negative (*T*. *cruzi*-Neg) serum samples. The established RI cut-off value was 1.0 (dashed line), with corresponding shaded areas representing grey zones (RI = 1.0 ± 0.10). Solid lines indicate geometric mean RI values and corresponding 95% CI values. AUC (Area Under Curve); Sen (Sensitivity); Spe (Specificity); Acc (Accuracy); *K* (*Kappa* index); CI (Confidence interval).

The performance of the IBMP-DAgS-ELISA was evaluated using a panel of 207 seropositive samples and 205 seronegative samples. Fig 2 illustrates reactivity index (RI) distributions and assay performance parameters obtained for each of the IBMP chimeras (individual data points are available in S1 Table). AUC (area under the curve) analysis revealed values above 98% for all four IBMP antigens, indicating the elevated overall capacity of these molecules to correctly identify positivity and negativity in serum samples. Due to overlap in 95% CI values, no differences were observed among AUC results. Mean RI values for *T*. *cruzi*-positive samples were significantly higher compared to *T*. *cruzi*-negative samples (p <0.0001; Fig 2). Considering *T*. *cruzi*-positive samples, a significant difference was observed between the highest RI value achieved using IBMP-8.3 (RI = 2.11) compared to the lowest RI value using IBMP-8.2 (RI = 1.64). No significance was observed when comparing among the other RI values obtained under IBMP-8.1 and IBMP-8.4. With respect to *T*. *cruzi*-negative samples, the lowest RI intensity was obtained for IBMP-8.2, followed by IBMP-8.3, IBMP-8.4 and IBMP-8.1.

The IBMP-8.1 protein misdiagnosed 53 (25.6%) *T*. *cruzi*-positive samples as negative, whereas IBMP-8.2 misdiagnosed 27 (13%) samples, IBMP-8.3 misdiagnosed 24 (11.6%) and IBMP-8.4 misdiagnosed 43 (20.8%) samples, resulting in sensitivity values of 74.4%, 87.0%, 88.4% and 79.2%, respectively (Fig 2). When taking 95% CI values into account, the IBMP-8.2 and IBMP-8.3 proteins correctly diagnosed more *T*. *cruzi*-positive samples than IBMP-8.1; however, no differences in 95% CI values were observed between the IBMP-8.4 protein and the other three molecules. Regarding specificity, all antigens offered 100% specificity except IBMP-8.3 (96.6%), which misdiagnosed 7 (3.4%) *T*. *cruzi*-negative samples as positive. No significant differences were seen with regard to specificity values. Considering IBMP-DAgS-ELISA accuracy, IBMP-8.2 offered the highest overall accuracy (93.4%), followed by IBMP-8.3 (92.5%), IBMP-8.4 (89.6%) and IBMP-8.1 (87.1%) (Fig 2). Accordingly, IBMP-8.2 was shown to be significantly more accurate compared to IBMP-8.1. Qualitative assessments using Cohen’s *kappa* coefficient revealed substantial agreement for IBMP-8.1 and IBMP-8.4 compared to previously obtained diagnostic results, and almost perfect agreement for IBMP-8.2 and IBMP-8.3 (Fig 2).

With respect to RI values ranging between 0.9 and 1.10, i.e., those falling in the grey zone (1.0 ±0.10), we observed that RI results from all *T*. *cruzi*-negative samples fell outside the grey zone when assayed using the IBMP-8.1, IBMP-8.2 and IBMP-8.4 proteins (Fig 2). Contrarily, the RI values of three negative samples (0.7%) returned inconclusive results when tested with the IBMP-8.3 protein. With respect to *T*. *cruzi*-positive samples, 4 (1.0%), 5 (1.2%), 16 (3.9%) and 25 (6.1%) fell in the grey zone when assayed with IBMP-8.2, IBMP-8.3, IBMP-8.1 and IBMP-8.4, respectively.

In an attempt to reduce diagnostic uncertainty, analyses involving series and parallel approaches were conducted using the results obtained from individual IBMP chimera DAgS-ELISA testing (Table 1). These approaches are strategies for combining two diagnostic test results. Sensitivity was observed to increase consistently when ELISA test results were analyzed in parallel, in comparison to individual chimera test values or using a series approach. We found that any combination of IBMP proteins offered higher sensitivity than when the chimeras were assayed individually, with the pair of IBMP-8.1+ IBMP-8.4 providing the lowest combined sensitivity (>94%). With respect to specificity, when samples were assayed using either IBMP-8.1, IBMP-8.2 or IBMP-8.4 individually, or in any combination of these three proteins analyzed both either series or parallel approaches, values reached 100%. Parallel analysis revealed that any combination of chimeras involving the IBMP-8.3 protein provided 96.6% specificity. Diagnostic accuracy as analyzed by the series approach revealed inferior accuracy with regard to most combinations compared to individual test results, except IBMP-8.2+ IBMP-8.3. Conversely, under parallel analysis, any IBMP test combination provided superior accuracy values compared to individual or series approach results.

**Table 1:** IBMP-DAgS-ELISA diagnostic test assay results compared both individually and under several combinations of IBMP chimeras; additional analysis performed using series and parallel approaches.

IBMP-DAgS-ELISA	Type	Sen (95% CI)	Spe (95% CI)	Acc (95% CI)
IBMP-8.1	Individual	74.4 (68.0–79.9)	100 (98.2–100)	87.1 (83.6–90.0)
IBMP-8.2	Individual	87.0 (81.7–90.9)	100 (98.2–100)	93.4 (90.6–95.5)
IBMP-8.3	Individual	88.4 (83.3–92.1)	96.6 (93.1–98.3)	92.5 (89.5–94.6)
IBMP-8.4	Individual	79.2 (73.2–84.2)	100 (98.2–100)	89.6 (86.2–92.2)
IBMP-8.1+ IBMP-8.2	Series	64.7 (55.6–72.6)	100 (99.7–100)	82.3 (77.7–86.3)
	Parallel	96.7 (94.1–98.2)	100 (96.4–100)	98.3 (95.3–99.1)
IBMP-8.1+IBMP-8.3	Series	65.8 (56.6–73.6)	100 (99.9–100)	82.8 (78.2–86.7)
	Parallel	97.0 (94.7–98.4)	96.6 (91.4–98.3)	96.8 (93.1–98.4)
IBMP-8.1+ IBMP-8.4	Series	58.9 (49.8–67.3)	100 (99.9–100)	79.4 (74.8–83.6)
	Parallel	94.7 (91.4–96.8)	100 (96.4–100)	97.3 (93.9–98.4)
IBMP-8.2+ IBMP-8.3	Series	76.9 (68.1–83.7)	100 (99.9–100)	88.4 (83.9–91.8)
	Parallel	98.5 (96.9–99.3)	96.6 (91.4–98.3)	97.5 (94.2–98.8)
IBMP-8.2+ IBMP-8.4	Series	68.9 (59.8–76.5)	100 (99.9–100)	84.4 (79.8–88.2)
	Parallel	97.3 (95.1–98.6)	100 (96.4–100)	98.6 (95.8–99.3)
IBMP-8.3+ IBMP-8.4	Series	70.0 (61.0–77.6)	100 (99.9–100)	84.9 (80.3–88.7)
	Parallel	97.6 (95.5–98.8)	96.6 (91.4–98.3)	97.1 (93.5–98.5)

Sen (Sensitivity); Spe (Specificity); Acc (Accuracy); CI (Confidence interval)

### Cross-reactivity analysis

**Fig 3:**
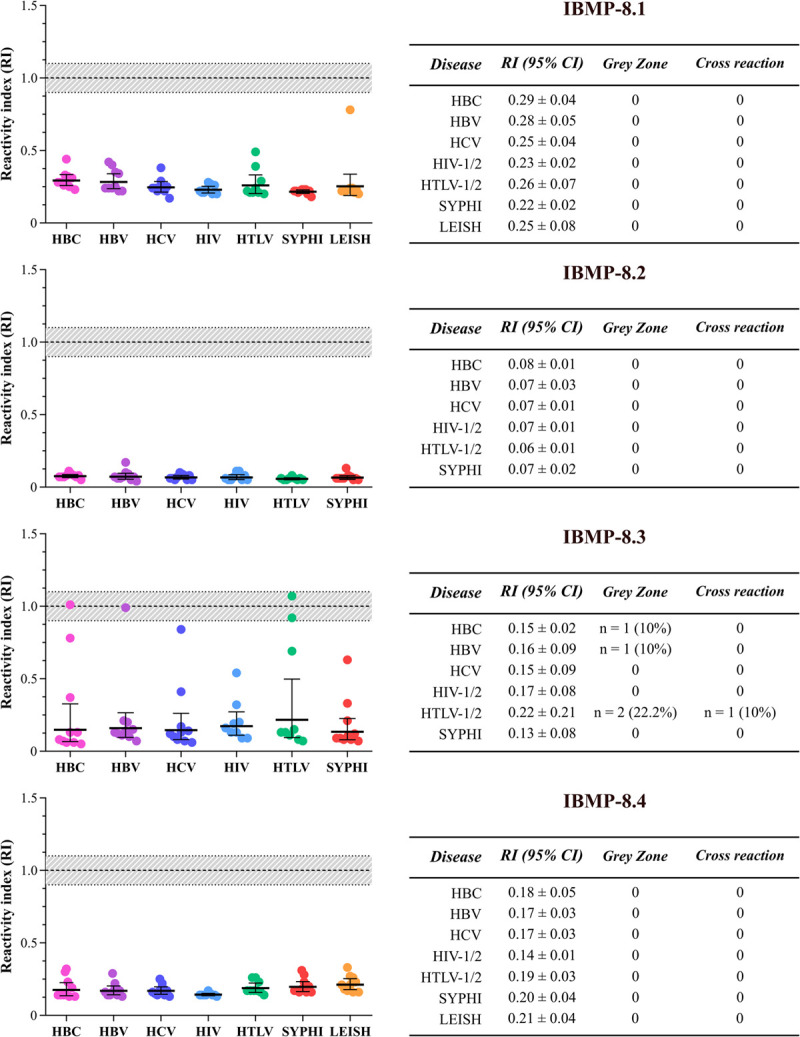
Analysis of cross-reactivity using four *Trypanosoma cruzi* chimeric proteins in sera from individuals with unrelated infections. **Reactivity index (RI) values for each infection are shown via dot-plots with corresponding 95% CI values.** The established RI cut-off value was 1.0 (dashed line), with corresponding shaded areas representing grey zones (RI = 1.0 ± 0.10). Solid lines indicate geometric mean RI values and corresponding 95% CI values. HBC (hepatitis B core); HBV (hepatitis B virus); HCV (hepatitis C virus); HIV (human immunodeficiency virus); HTLV (human T-cell lymphotropic virus); SYPHI (syphilis); LEISH (visceral leishmaniasis).

IBMP-DAgS-ELISA was also performed to assess antigenic cross-reactivity against antibodies of unrelated infections (RI ≥ 1.0) using a panel of 68 serum samples (Fig 3; individual data points are available in S2 Table). No cross-reactivity or inconclusive results were obtained when these samples were assayed using IBMP-8.1, IBMP-8.2 or IBMP-8.4. On the other hand, the results from 4 samples (HBC = 1; HBV = 1; HTLV-1/2 = 2) fell inside the grey zone when assayed using the IBMP-8.3 protein. Cross-reactivity (RI = 1.07) was identified using IBMP-8.3 in one of the HTLV-1/2-positive samples that fell in the grey zone. Due to limited availability of samples, *Leishmania*-positive samples were tested only with IBMP-8.1 and IBMP-8.4 because they had lower cross-reactivity values in previous studies [9,13].

## Discussion

As DAgS-ELISA is not limited to the detection of class-specific antibodies, this assay technique has been shown to offer increased sensitivity [19,25]. It follows that the use of this diagnostic approach may be useful in the detection of anti-*T*. *cruzi* antibodies in both humans and mammalian hosts, since secondary antibodies are not used as detection agents. Importantly, no commercial tests are currently available to detect anti-*T*. *cruzi* antibodies in mammalian hosts. In addition, the absence of background signals in negative samples has been reported in DAgS-ELISA [21,25] with high diagnostic test sensitivity since there is no need to dilute samples. The present study constitutes a phase I study (proof-of-concept) that verified the significant capability of four IBMP chimeric proteins to differentiate between *T*. *cruzi*-positive and -negative samples.

Indeed, high AUC values reflecting diagnostic accuracy were obtained using all four IBMP antigens. Although the AUC values reported herein are lower than those described using other previously evaluated diagnostic platforms, e.g., indirect ELISA [12,13], liquid microarray [14], lateral flow assay [16] and surface plasmon resonance [17], IBMP-DAgS-ELISA demonstrated high diagnostic capacity, achieving values above 95.8%. Moreover, mean signal values as measured by RI were significantly higher in *T*. *cruzi*-positive samples compared to negative samples. Surprisingly, IBMP-8.2 demonstrated higher AUC (99.5%) and accuracy (93.4%) values, while IBMP-8.4, the protein with the largest repertoire of epitopes [12], offered just 79.2% sensitivity. In all previous evaluations of diagnostic performance, IBMP-8.4 attained over 99% sensitivity, compared to variable results for IBMP-8.2 (between 94.3–98.2%) [12,13,15,35]. The observed discrepancies may be explained by the size and molecular weight of these two molecules, as IBMP-8.4 contains a higher number and variety of epitopes compared to IBMP-8.2, which could induce steric hindrance during the formation of immune complexes. Another possibility for discrepancy in sensitiviy values worth considering is the prolonged period (two years) between when the IBMP antigens were conjugated to peroxidase and the time of analysis of DAgS-ELISA performance. Although all conjugated antigens were kept frozen throughout this entire period, it is impossible to guarantee the stability of the conjugated antigens for this length of time, which could lead to false-negative results, thereby leading to decreased test sensitivity.

The discrepancy in sensitivity values can also be explained by the different amino acid composition of IBMP antigens. It is already known that conjugation of HRP with antibodies/antigens occurs at the -NH2 (amine) group of a lysine or at the free -SH (sulfhydryl) group of a cysteine [36]. Immunoglobulin G (IgG), for example, has an average of about 80 lysine residues, of which 20 (~25%) are present at solvent-accessible sites. Conversely, cysteine residues are present in smaller numbers, although they are evenly distributed in the amino acid sequence: about 16 pairs of cysteine residues are present in the form of 12 (~75%) intra-chain and four inter-chain disulfide bonds. Sometimes inter-chain cysteine is selected for conjugation because it is accessible to solvents [37]. Therefore, 25% of the lysine and cysteine residues of IgG antibodies are available for conjugation with HRP. When these residues are close to each other, this impairs the conjugation of HRP at one of the binding sites by steric hindrance. In contrast to immunoglobulin G, chimeric IBMP antigens have only free amines of a lysine in varying amounts: IBMP-8.1 (8 residues), IBMP-8.2 (14 residues), IBMP-8.3 (32 residues), and IBMP-8.4 (27 residues). Since all molecules use specific spacers between epitopes to prevent protein folding and to hide antibody binding sites [12], the number of lysine residues available for binding to HRP is probably larger than the number estimated for IgG (~25%). Therefore, IBMP-DAgS ELISA sensitivity values found here could be influenced by the number of lysine residues available for binding to HRP. Indeed, the molecule with more lysine residues had the highest sensitivity value (88.4%). The lysine residues in IBMP-8.3 antigen are evenly distributed throughout the protein, suggesting that most (if not all) residues bind to HRP. Therefore, it is likely that the binding of HRP to IBMP-8.3 is not affected by stereochemical hindrance. Interestingly, IBMP-8.4, a molecule with 27 lysine residues, had a lower sensitivity value (79.2%) than IBMP-8.2 (87.0%), which has only 14 lysine residues. Contrasted to IBMP-8.3 and IBMP-8.2, the distribution of lysine residues in the IBMP-8.4 molecule is not homogeneous, which may lead to steric hindrance and affect the peroxidase conjugation process. Finally, IBMP-8.1 had the lowest sensitivity value (74.4%) among the four molecules, which is probably due to the low number of lysine residues. For the present study, the molar ratio between IBMP antigens and HRP was set at 1:1. Because the lysine residues are different among IBMP antigens, other molar ratios will be considered in future studies.

IBMP-DAgS-ELISA was shown to be a high specificity assay, as only one antigen (IBMP-8.3) was not found to be 100% specific. Our data stand in accordance with the results from previous studies employing indirect ELISA [12,13,15,35,38] as well as other diagnostic platforms [14,16,17]. Serological cross-reactivity using the IBMP proteins was an unsurprising finding, as weak seropositivity in other infectious diseases was detected in previous evaluations [13–15]. Importantly, while four samples fell inside the grey zone when assayed using IBMP-8.3, no cross-reactions or grey zone results were observed for IBMP-8.1, IBMP-8.2 or IBMP-8.4. This was not surprising, as IBMP-8.3 showed the lowest specificity and was the only antigen that had high RI values for *T*. *cruzi*-negative samples. The RI values found under cross-reactivity analysis were lower than those reported using indirect ELISA [13,15], which could be attributable to the absence of a background signal in negative samples, as has been similarly reported by other authors [21,25]. Due to limited sample availability, cross-reactivity evaluations involving *Leishmania* spp., were unfortunately limited to IBMP-8.1 and IBMP-8.4; no instances of cross-reactivity were observed for these two proteins, which stands in agreement to a previous report from our group [9]. Our previous results indicated the absence of any cross-reactivity using IBMP-8.4 in samples of American cutaneous (n = 575) and visceral (n = 172) leishmaniasis. Similarly, no cross-reactivity and weak seropositivity (0.7%; 4/575) were described for IBMP-8.1 using cutaneous and visceral leishmaniasis, respectively [9]. Serological cross-reactivity against *Leishmania* spp. will be investigated in a phase II study with a larger sample size using IBMP-8.2 and IBMP-8.3.

Our study is limited by the fact that there was a longer period of time between the conjugation of the IBMP antigens to the peroxidase and the performance of the DAgS-ELISA assay, which may have influenced the low sensitivity values observed here. In addition, the interpretation of the present results is limited by the low number of sample available for cross-reactivity analysis. Importantly, these two limitations will be addressed in a future phase II study. The stability of the labeling process will be evaluated in a future study using a new batch of peroxidase-labeled antigens in different molar ratios (HRP: IBMP), and the results will be compared with both commercial indirect ELISA and the results presented here. Nonetheless, the findings presented herein demonstrate that all four IBMP chimeric proteins offer high overall diagnostic capability (AUC ≥ 98.6%).

Our findings suggest that IBMP-DAgS-ELISA is suitable for the detection of anti-*T*. *cruzi* antibodies in areas of co-endemicity with *Leishmania* spp. The findings also demonstrate the notable capability of all four IBMP proteins to distinguish between *T*. *cruzi*-positive and -negative samples. The specificity attained under DAgS-ELISA reached 100% using three of the four chimeric antigens evaluated, IBMP-8.1, IBMP-8.2 and IBMP-8.4. The data reported herein provide strong support for the eligibility of all four IBMP recombinant chimeric proteins to enter phase II studies. Future studies will also consider other mammalian reservoirs.

## Supporting information

S1 Checklist STARD checklist. (PDF)

S1 Table Reactivity index values for diagnostic performance assessment. (XLSX)

S2 Table Reactivity index values for cross-reactivity assessment. (XLSX)
